# SNPs for Parentage Testing and Traceability in Globally Diverse Breeds of Sheep

**DOI:** 10.1371/journal.pone.0094851

**Published:** 2014-04-16

**Authors:** Michael P. Heaton, Kreg A. Leymaster, Theodore S. Kalbfleisch, James W. Kijas, Shannon M. Clarke, John McEwan, Jillian F. Maddox, Veronica Basnayake, Dustin T. Petrik, Barry Simpson, Timothy P. L. Smith, Carol G. Chitko-McKown, and the International Sheep Genomics Consortium

**Affiliations:** 1 U.S. Meat Animal Research Center (USMARC), Clay Center, Nebraska, United States of America; 2 Department of Biochemistry and Molecular Biology, School of Medicine, University of Louisville, Louisville, Kentucky, United States of America; 3 Division of Animal, Food and Health Sciences, CSIRO, Brisbane, Australia; 4 AgResearch, Invermay Agricultural Center, Mosgiel, New Zealand; 5 Meat and Livestock Australia, North Sydney, Australia; 6 GeneSeek, a Neogen company, Lincoln, Nebraska, United States of America; University of Sydney, Australia

## Abstract

DNA-based parentage determination accelerates genetic improvement in sheep by increasing pedigree accuracy. Single nucleotide polymorphism (SNP) markers can be used for determining parentage and to provide unique molecular identifiers for tracing sheep products to their source. However, the utility of a particular “parentage SNP” varies by breed depending on its minor allele frequency (MAF) and its sequence context. Our aims were to identify parentage SNPs with exceptional qualities for use in globally diverse breeds and to develop a subset for use in North American sheep. Starting with genotypes from 2,915 sheep and 74 breed groups provided by the International Sheep Genomics Consortium (ISGC), we analyzed 47,693 autosomal SNPs by multiple criteria and selected 163 with desirable properties for parentage testing. On average, each of the 163 SNPs was highly informative (MAF≥0.3) in 48±5 breed groups. Nearby polymorphisms that could otherwise confound genetic testing were identified by whole genome and Sanger sequencing of 166 sheep from 54 breed groups. A genetic test with 109 of the 163 parentage SNPs was developed for matrix-assisted laser desorption/ionization–time-of-flight mass spectrometry. The scoring rates and accuracies for these 109 SNPs were greater than 99% in a panel of North American sheep. In a blinded set of 96 families (sire, dam, and non-identical twin lambs), each parent of every lamb was identified without using the other parent’s genotype. In 74 ISGC breed groups, the median estimates for probability of a coincidental match between two animals (P_I_), and the fraction of potential adults excluded from parentage (P_E_) were 1.1×10(−39) and 0.999987, respectively, for the 109 SNPs combined. The availability of a well-characterized set of 163 parentage SNPs facilitates the development of high-throughput genetic technologies for implementing accurate and economical parentage testing and traceability in many of the world’s sheep breeds.

## Introduction

Significant gains in efficiency are realized in production systems that use teams of rams for breeding ewes. These advantages include fewer enclosures and equipment, reduced labor, and increased mating efficiency. However, the success of genetic evaluations systems is directly affected by the accuracy of pedigrees. Misidentification of parents reduces the genetic gain and is economically disadvantageous [1], [2]. Parentage can be accurately determined in livestock with the use of single nucleotide polymorphisms (SNPs). These DNA markers have been used extensively to determine parentage in cattle [3]–[7] and have been proposed for use in sheep [8].

There are numerous theoretical approaches for DNA-based parentage assignment. These range from simple exclusion, to categorical and fractional allocation, to genotype reconstruction [9], [10]. The present report focuses on parentage exclusion as it is the least complicated method of parentage analysis. The approach is based on the principle that a parent and offspring must share an allele at every locus [11] and the probability of exclusion (P_E_) is the probability that an alleged parent would be excluded from parentage [5], [12], [13]. This simple approach requires high genotyping accuracy (≥99%) and high minor allele frequency (MAF, ≥0.30). Thus, selecting SNPs with these qualities in many breeds is critical for successful parentage assignment in flocks around the world.

An important and challenging use of SNP parentage testing is the assignment of one parent without knowledge of the other parent’s genotype. For this application, a candidate parent with a homozygous genotype is excluded when the offspring has the opposing homozygous genotype. Achieving accurate parentage assignment without the other parent’s genotype, while also keeping the number of SNPs (i.e., cost) to a minimum, requires that each “parentage SNP” has a high P_E_ value in as many breeds as possible. SNPs with high P_E_ values also tend to have a low probability of identity (P_I_) values, i.e., the probability that two animals selected at random from the same population would have identical genotypes [13], [14]. Thus, parentage SNPs are also ideal as unique molecular identifiers for tracing sheep products to their source.

Four sets of parentage SNPs have been used with Australian and New Zealand sheep since the Ovine SNP50k BeadArray was reported by the International Sheep Genomics Consortium (ISGC) [15]. The autosomal parentage SNPs in these sets include: 84 and 300 from New Zealand’s AgResearch [16], 88 from the ISGC [17], and 382 from Australia’s Commonwealth Scientific and Industrial Research Organisation (CSIRO) and Sheep Cooperative Research Centre (SheepCRC) [18]. A minimal overlapping set of highly informative SNP markers that are suitable for use in globally diverse breeds of sheep would be beneficial for achieving high overall genotyping efficiency and economy of scale.

The present article describes the identification and characterization of 163 parentage SNPs with the exceptional overall qualities for use in diverse breeds of sheep, and a subset of 109 parentage SNPs developed for use in North American sheep. Of these 109 parentage SNPs, 34, 44, 55, and 56 were also contained within the four international parentage sets, respectively, and thus provide reference markers for standardization between sets. The set of 109 parentage SNPs also contained 22 that had not previously been identified or used in any parentage SNP set. The combined power of the 109 SNPs for determining parentage and tracing animals appeared to be suitable for use in many breeds throughout the world.

## Results

### Identification and Characteristics of 163 Ovine Parentage SNPs

**Figure 1:**
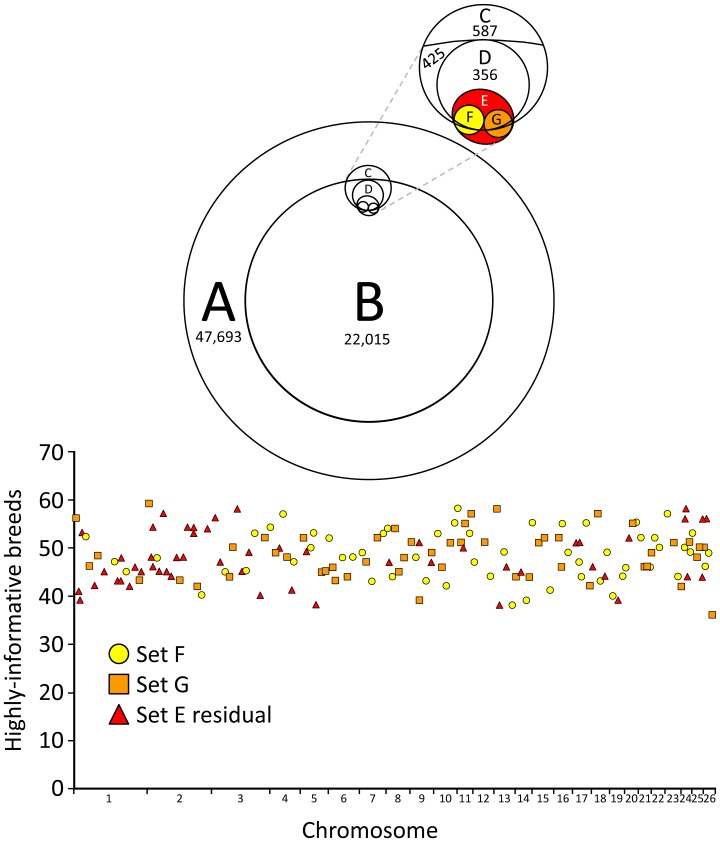
Venn diagram of SNP sets in this study and genome distribution of 163 parentage SNPs. Venn diagram: Set A, autosomal SNPs from the Ovine SNP50k Bead Array; Set B, SNPs with a MAF greater than or equal to 0.3 in at least 36 of the 74 ISGC breed groups; Set C, SNPs from four preexisting ovine parentage SNP panels (425 inside set B); Set D, SNPs with MAF greater than or equal to 0.3 in a U.S. sheep panel; Set E, 163 USDA parentage SNPs from the present report with 42 outside Set C; Sets F and G, 57 and 52 USDA parentage SNPs used in two respective multiplex assays developed for use in North American sheep (12 and 17 SNPs outside Set C, respectively). **Graph:** distribution of 163 parentage SNPs across 26 autosomal chromosomes. A SNP was classified as highly-informative in a breed if the MAF was greater or equal to 0.3.

Starting with 47,693 autosomal SNPs on the Ovine SNP50 Bead Array, markers were evaluated in a step-wise fashion by multiple criteria to identify those with desired properties (Table 1). A SNP was defined as highly informative in a breed group if its MAF was greater than or equal to 0.3. There were 22,015 SNPs that were highly-informative in at least 36 of the 74 ISGC breed groups (Figure S1). The set of 22,015 SNPs was compared with the set of 587 unique SNPs from four ovine parentage SNP panels to identify 425 SNPs in the intersection (Figure 1, sets B and C). There were 356 of the 425 SNPs that were also highly informative in a 96-member panel of diverse U.S. sheep (Figure 1, set D). The context sequences of the 356 candidate SNPs were evaluated by analyzing whole genome sequence (WGS) from 75 ISGC sheep and Sanger sequence from the 96 U.S. sheep. Of the 356 candidate SNPs, 235 (66%) were dismissed because the context sequences had one or more intrinsic molecular properties that negatively impact SNP testing (listed in Table 1). The remaining 121 parentage SNPs were augmented with 42 additional markers selected with the same criteria, but not previously from an ovine parentage SNP panel. These 163 parentage SNPs (Figure 1, set E) were further evaluated as a group.

**Table 1:** Parentage SNP selection criteria for use in globally diverse breeds of sheep.

Criteria	Benefits
Concurrent membership on OvineSNP50 Bead Array (autosomes)	Increased standardization
Highly informative^a^ in at least 36 ISGC breed groups and a U.S. sheep panel^b^	Increased P_E_ and P_I_
Concurrent membership in any of four parentage SNP sets^b^	Increased standardization
Only two nucleotide alleles observed	Improved assay design
Not part of an insertion or deletion polymorphism	Increased testing accuracy
Absence of large blocks of repetitive DNA nearby	Increased quality control^c^
Unique map location	Increased testing accuracy
Even distribution of parentage SNPs (approximately 15 Mb)	Reduced allelic association
Nearby polymorphisms identified in 166 sheep and 50 breeds^d^	Increased testing accuracy
Parentage SNP region correctly amplified by PCR in a U.S. sheep panel and verified by Sanger sequencing	Increased testing quality control
Consistent Mendelian inheritance patterns in 95 tetrad families^e^	Increased test validation

a MAF greater than or equal to 0.3 in the specified group.

b See Materials and Methods for description of sets.

c Large blocks of repeats (>1 kb) in nearby sequence precludes the production of unique 750 bp PCR fragments for Sanger sequencing, and thus hinders independent validation of genotypes.

d Nearby SNPs and indels identified within approximately 350 bp of the parentage SNP.

e Described in Materials and Methods.

**Figure 2:**
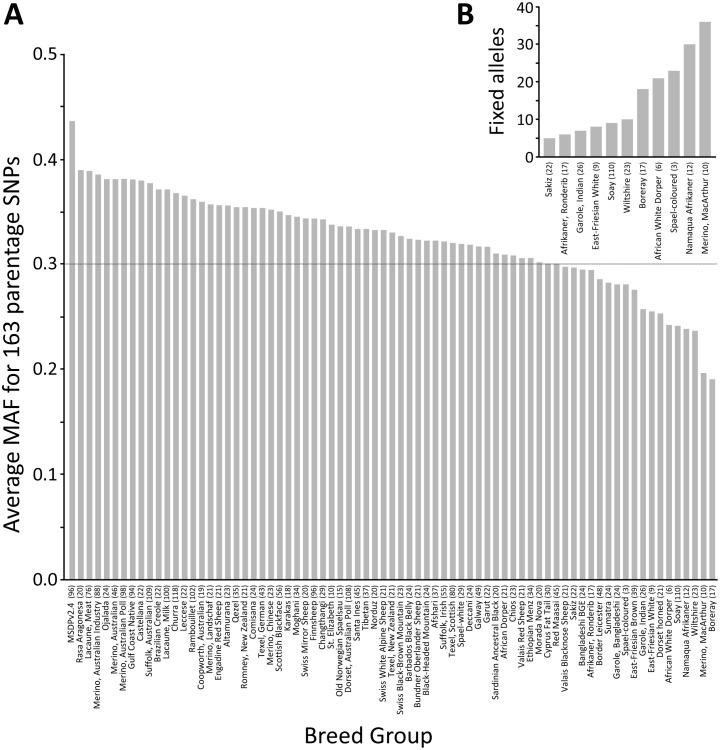
Average MAFs for the set of 163 parentage SNPs by ISGC breed group. **Panel A:** average MAFs for 163 parentage SNPs by breed group. MSDPv2.4 is the USMARC Sheep Diversity Panel version 2.4 (Materials and Methods). The number in parentheses for each breed group is the number of animals used. **Panel B:** breeds with five or more parentage SNPs having with fixed alleles (i.e., MAF = 0).

**Figure 3:**
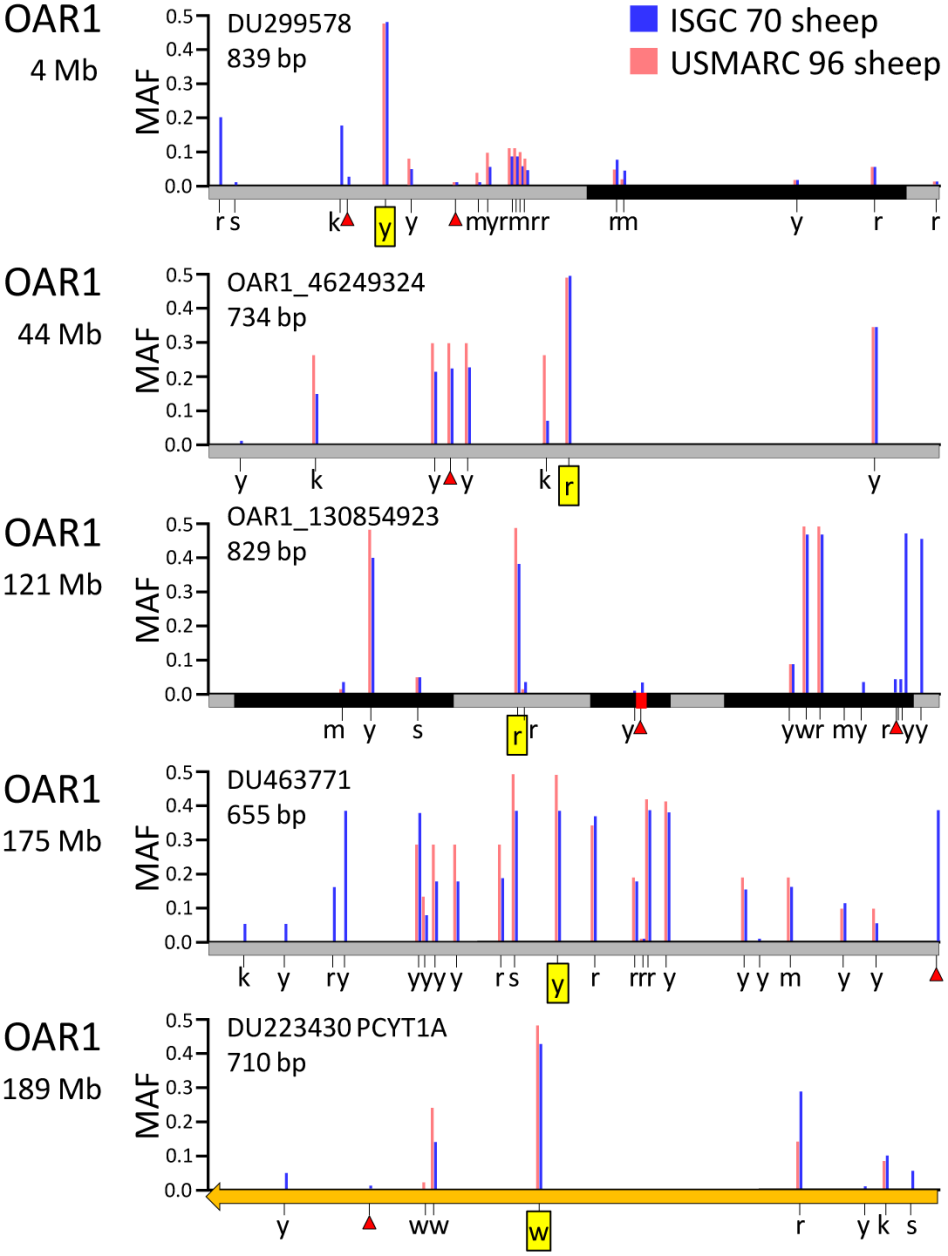
Physical maps of five representative amplicons with parentage SNPs. High resolution map of five regions on ovine chromosome 1 that were targeted for *in silico* NGS analysis and PCR-amplification for Sanger sequencing and analysis. The parentage SNP is boxed in yellow. SNP positions are indicated by blue and red vertical bars and denote frequency of SNPs in an international panel of 70 sheep and a panel of 96 U.S. sheep, respectively and IUPAC/IUBMB ambiguity codes for nucleotides (r = a/g, y = c/t, m = a/c, k = g/t, s = c/g, w = a/t) [35]. Other symbols: red triangles, indel polymorphisms; black rectangles, repetitive elements grey rectangles, intergenic regions; orange arrows, exons.

The 163 parentage SNPs were, on average, highly informative in 48 of the ISGC breed groups (±5.1). The average MAF for 163 SNPs across all 74 breed groups was 0.33±0.04. The names, MAFs, GenBank accession numbers, and other features of the 163 parentage SNPs are provided in Table S1. In addition, a search of GenBank’s nucleotide database with the terms “USMARC sheep parentage” retrieves links to all 163 accession files. The Rasa Aragonesa and Boreray breed groups had the highest and lowest within-breed MAFs, respectively (0.40 and 0.20, Figure 2A). In each breed group, only a few SNPs were uninformative. There were 63 breed groups that had three or less parentage SNPs with MAFs of zero (Table S2). Conversely, the MacArthur Merino breed group had 36 SNPs that were apparently fixed for one allele, based on a sample of 10 sheep (Figure 2B). The average intra-chromosomal distance between parentage SNPs was 15.3 Mb±7.1 (Table S1). Analysis of WGS or Sanger sequence in 166 sheep from 54 breed groups identified 2,917 nearby polymorphisms and 330 repetitive DNA elements in the regions immediately surrounding the parentage SNPs. Five representative examples of parentage SNPs regions with these features are shown in Figure 3. Knowledge of these features provided a guide for designing oligonucleotides for Sanger sequencing and matrix-assisted laser desorption/ionization–time-of-flight mass spectrometry (MALDI-TOF MS) assays. PCR primers and assay probes were designed to hybridize with unique sequences that are highly conserved in most breeds. Together, these results provide information necessary and sufficient for automated or manual assay design on a variety of genotyping platforms.

### MALDI-TOF MS Assay with 109 SNPs for use on U.S. Sheep

**Figure 4:**
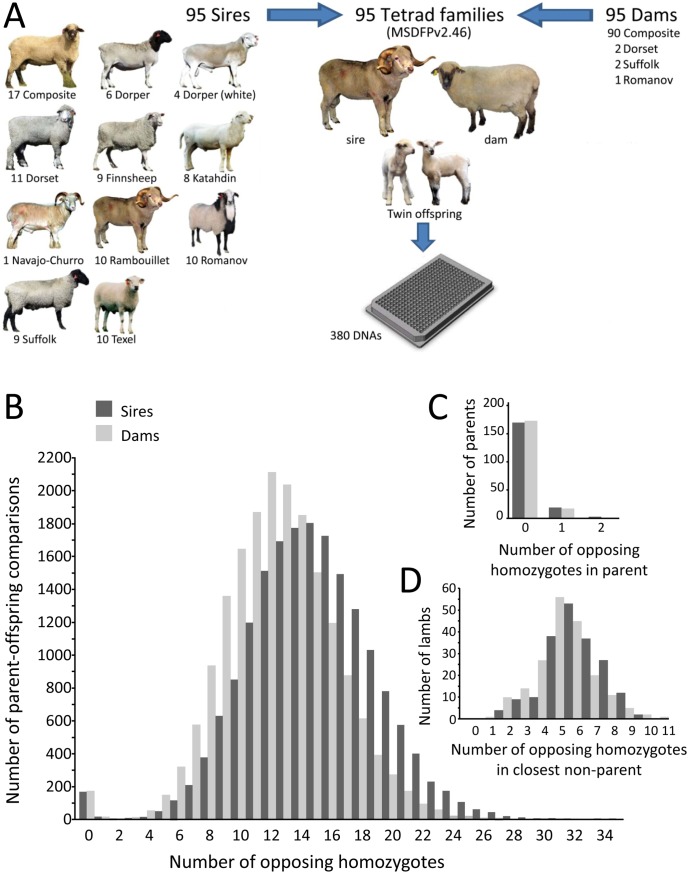
Parentage exclusion in 95 tetrad families with 109 parentage SNPs. **Panel A:** Structure of the USMARC Sheep Diversity Family Panel version 2.46. **Panel B:** Distribution of the opposing homozygous SNPs genotypes in a pair-wise comparison of all possible combinations of parents and offspring (36,864 comparisons between an adults and an offspring). **Panel C:** Distribution of opposing homozygotes between the true parents and offspring (380 comparisons between lambs and sires/dams). **Panel D:** Distribution of the opposing homozygotes in a pair-wise SNP comparison of the 190 lambs and 95 each of the closest matching ram and ewe that were not parents of the lambs (380 comparisons between lambs and rams/ewes).

Multiplex assays of 57 and 52 SNPs were developed starting from the 163 parentage SNPs, as described in the Materials and Methods. In the first round of development, 119 parentage SNPs were selected for testing and 117 markers produced quality genotypes in a U.S. panel of 96 rams (assay conversion rate 98.3%). Comparison of these genotypes with those derived from Sanger Sequencing and the OvineSNP50 Bead Array indicated that eight of the 117 SNPs did not meet the cutoffs for 97% scoring rate (i.e. “call rate”) and 99% accuracy (data not shown). These eight MALDI-TOF MS assays were omitted from subsequent rounds of MALDI-TOF MS multiplex assay development. The remaining MALDI-TOF MS assays for 109 parentage SNPs (multiplexes of 57 and 52 SNPs) were used to genotype 95 tetrad families (Figure 4A). Thirteen of 380 animals each had an average SNP call rate of less than 97% on the first pass and were typed a second time together with 35 previous samples to fill out a 96-element quadrant (i.e., 48 samples with two multiplexes each). This practice is common in a commercial genotype-production setting. Subsequent scoring and analysis were derived directly from the combined data sets of 332 animals genotyped once and 48 animals genotyped twice.

In the U.S. panel of 96 rams, there were 10,464 SNP genotypes possible from the 109 SNPs and 10,452 of them were reported (99.89% SNP call rate). When the 10,452 MALDI-TOF MS genotypes were compared to those from Sanger sequencing and the Ovine SNP50k Bead Array, 10,431 were in agreement and 21 were discordant owing to MALDI-TOF MS genotyping errors (99.80% SNP accuracy). In the set of 380 sheep from the 95 tetrad families, there were 41,420 genotypes possible from the 109 SNPs and 41,037 of them were reported (99.08% SNP call rate). The SNP call rate for complete tetrad families was also relevant for parentage analysis (i.e., the SNP call rate for all four members of the family). In the 95 tetrad families, genotypes from all four family members were reported in 10,061 of the 10,355 possible cases (97.16% tetrad family call rate). In these 10,061 sets of tetrad genotypes, there were 10,047 inheritance patterns that were consistent with Mendelian expectations (99.86%).

### Parentage Assignment with 109 SNPs in Families of U.S. Sheep

The 95 tetrad families were used to evaluate the use of the 109 parentage SNP MALDI-TOF MS assays for assigning exactly two parents to each offspring without using genotypes from the other parent. These families had germplasm derived from 11 breeds of sheep (Figure 4A). A noteworthy feature of the 95 sires was the diversity of breeds and lack of shared grandparents. However, among the 95 ewes, 52 ewes shared 0.25 of their genome with other ewes in the same group. In “one-parent” parentage testing, a candidate parent was excluded when the candidate and the offspring had opposing homozygous SNP genotypes. Pair-wise genotype comparisons were made for all possible combinations of 190 parents and 190 offspring with 109 SNPs (i.e., 36,100 total parent-offspring pairs and 3,934,900 possible SNP genotype comparisons). Assuming the SNP call rate of 99.08% for the parents and the offspring, there were approximately 3.9 million pair-wise SNP genotype comparisons made. The number of opposing homozygous SNP genotypes appeared to be normally distributed with the peaks centered at 15 and 12 for the sires and dams, respectively (Figure 4B). For each lamb, the true sire and dam were correctly identified as those having the fewest exclusions (Figure 4C). In cases where genotyping error caused spurious opposing homozygous genotypes between the true parent and the offspring, the next closest candidate for parentage still had more opposing homozygous genotypes. On average, the next closest candidate for sire and dam had 6 and 5 opposing homozygous genotypes, respectively (Figure 4D). Although purebred families with closely related sires were not available for the most stringent test of determining parentage, this MALDI-TOF MS multiplex assay with 109 parentage SNPs was efficient and accurate in this sample of sheep.

### P_I_ and P_E_ in Globally Diverse Breeds of Sheep

For each of the 74 ISGC breed groups, the P_I_ and P_E_ for each SNP was estimated from its genotype and allele frequencies as described in the Materials and Methods (Table S2). Although 163 parentage SNPs were available for analysis, a more realistic scenario was to estimate P_I_ and P_E_ for the 109 SNPs used in the MALDI-TOF MS assay. The median within-breed P_I_ estimated for the combined set of 109 parentage SNPs was 1.1×10^−39^ (Afshari, Table S2). The within-breed maximum and minimum P_I_ were 1.5×10^−25^ (Boreray) and 9.3×10^−45^ (Gulf Coast Native), respectively. Thus, for the purposes of traceability, it would be unlikely that two samples with matching genotypes would be from different animals.

The P_E_ was estimated under stringent conditions where genotype information from only one parent was used (i.e., “one-parent parentage”). Among unrelated candidate parents, the median within-breed P_E_ for the combined 109 parentage SNPs was estimated to be 0.999987 (Swiss White Alpine Sheep, Table 2). Although the range of P_E_ for various breeds spanned four orders of magnitude, from Brazilian Creole (0.9999998) to the Namaqua Africaner (0.992), there was no apparent geographic bias. Assuming unrelated parents, breed groups within 0.000005 of the median P_E_ were from Australia, Bangladesh, Brazil, China, Germany, Indonesia, Iran, Jamaica, Spain, Sumatra, Switzerland, and the United Kingdom. As expected, close relationships between candidate parents reduced the P_E_ by two to six orders of magnitude, depending on the breed and the relationship (Table S2). Nevertheless, this subset of 109 parentage SNPs (and similar sets) are predicted to be useful in many globally diverse breeds of sheep.

**Table 2:** Statistics for the combined (1-PE) with one parent, by SNP set, and relatedness.

		*r* a			
SNP set and statistic^b^	Breed	0.000	0.125	0.250	0.500
163 SNPs					
Median	Australian Poll Dorset	8.0×10^−8^	6.8×10^−7^	5.7×10^−6^	3.6×10^−4^
Maximum	Brazilian Creole	6.0×10^−11^	1.4×10^−9^	3.2×10^−8^	1.3×10^−5^
Minimum	Macarthur Merino	2.8×10^−4^	8.5×10^−4^	2.5×10^−3^	2.0×10^−2^
109 SNPs^c^					
Median	Swiss White Alpine Sheep	1.3×10^−5^	5.7×10^−5^	7.3×10^−4^	2.9×10^−2^
Maximum	Brazilian Creole	1.3×10^−7^	1.1×10^−6^	4.3×10^−5^	7.5×10^−3^
Minimum	Namaqua Africaner	7.6×10^−3^	1.5×10^−2^	4.5×10^−2^	2.2×10^−1^
57 SNPs (MP1)^d^					
Median	Ethiopian Menz	3.7×10^−3^	7.7×10^−3^	2.7×10^−2^	1.7×10^−1^
Maximum	Brazilian Creole	3.2×10^−4^	9.4×10^−4^	5.9×10^−3^	8.3×10^−2^
Minimum	Namaqua Africaner	1.6×10^−1^	2.0×10^−1^	3.1×10^−1^	5.6×10^−1^
52 SNPs (MP2)					
Median	Tibetan	3.5×10^−3^	7.5×10^−3^	2.7×10^−2^	1.7×10^−1^
Maximum	Brazilian Creole	4.2×10^−4^	1.2×10^−3^	7.2×10^−3^	9.1×10^−2^
Minimum	East Friesian White	1.1×10^−1^	1.5×10^−1^	2.4×10^−1^	5.0×10^−1^

a Relatedness coefficient, i.e., the average proportion of genome shared between possible parents.

b Statistics were calculated for each of the 74 breed groups (Table S2). The P_E_ was calculated assuming the genotyping error rate was negligible, and only one parent was available (i.e., P_E_ = 2(χ_11_)(χ_22_)).

c The 109 SNP set is a specific subset of the 163 SNP set (Table S1) and used in MALDI-TOF MS assays.

d The 57 and 52 SNP sets are specific multiplex combinations of the 109 SNP set (Table S1).

### Evaluating the Accuracy of the P_E_ Estimate with 95 Families and 109 SNPs

The accuracy of the P_E_ estimate for a SNP was evaluated by comparing the measured frequency of opposing homozygous genotypes (i.e., the measured P_E_) to the predicted frequency of opposing homozygotes derived from the average genotype frequencies (i.e., the predicted P_E_). In this analysis, no correction was made for any family relationships among the parents. The average measured P_E_ was 0.113±0.037 for comparisons of 190 adults with the 190 offspring in the 95 tetrad families. The average predicted P_E_ calculated for 109 SNPs in the same 380 members of the 95 tetrad family panel (i.e. a random adult and a random offspring) was 0.129±0.021. The average difference between the measured P_E_ and the predicted P_E_ of a SNP was 0.016±0.033. Thus, the predicted P_E_ indicated that 1.6% more parents would be excluded by each SNP than were actually excluded. This likely reflects the impact of family relationships between some of the ewes.

## Discussion

This report describes the identification of 163 SNPs with exceptional qualities for use in parentage testing and traceability in globally diverse breeds of sheep. The application of stringent selection criteria identified SNPs that have a high degree of informativity and are amenable to accurate scoring by a variety of genotyping technologies. These SNPs are relatively unencumbered with negative attributes such as indels, repetitive structures, and unknown flanking SNPs, and thus more likely to perform well when interrogated by present and future genotyping technologies. A subset of 109 SNPs was also developed for a MALDI-TOF MS platform and used with success in “one-parent” parentage testing in U.S. sheep breeds. All 163 SNPs and the multiplex MALDI-TOF MS assays for the subset of 109 SNPs are available for world-wide use without restriction. Alternatively, other subsets from the 163 SNPs could be tailored to specific breeds and still have substantial overlap with existing SNP sets. If needed, more SNPs with similar properties could be developed and added. However, genetic linkage between SNP alleles increases as their distance decreases. The current average distance between the 163 SNP markers (15.3 Mb) is already small enough that a significant degree of haplotype sharing is expected between breeds [15]. Thus, the benefit of developing additional SNPs for use in parentage may be somewhat diminished with the accession of each new marker.

Until recently, commercial and research laboratory parentage testing in sheep was accomplished with sets of eight to 24 multi-allelic simple tandem repeat markers (i.e., microsatellites) [19]–[21]. An international panel of 12 microsatellites and a sex specific marker have been recommended by the International Society for Animal Genetics (ISAG) for use in their DNA comparison tests [22]. Accuracy, exclusion power, and standardization are among the top challenges for laboratories using any parentage marker set, including those with microsatellites. Genotype accuracy with the ISAG sheep microsatellite markers varied between laboratories, with 50% of those tested having error rates greater than 5% [22]. One source of microsatellite genotyping error comes from difficulties in standardizing microsatellite fragment lengths between genotyping systems. This is not an issue for SNP genotypes which can be reported as a single letter. SNPs are the fundamental unit of genetic variation in sheep and attractive as parentage markers because they are abundant [15], genetically stable [23], [24], and amenable to accurate high-throughput automated genotyping platforms [25]. As genotyping technologies continue to improve and the costs decrease, parentage testing is becoming more affordable. Despite the current cost of sheep microsatellite parentage tests, tens of thousands have been used worldwide to ensure pedigree accuracy and thereby increase the rate of genetic gain in sheep breeding programs. A typical microsatellite parentage test can be purchased for 25 to $35 US per animal. SNP tests with approximately 100 markers can be purchased for 15 to $20 US, and be reliably used for both parentage testing and tracing diseased animals to their source. For all of these reasons, SNPs have become the focus of efforts to improve sheep parentage testing.

Several factors may reduce the chances of success when applying the present results to other breeds and real world production settings. Inaccurate P_E_ estimates, poor quality assay designs, inefficient genotyping platforms, or degraded DNA samples from the field could result in parentage tests without sufficient discriminatory power. Ultimately, the usefulness of any set of parentage SNPs in a given population is determined locally by those who use it. The present report describes a commercial test for U.S. sheep that shares significant overlap with other contemporaneous international tests and provides a starting point and a rationale for designing other sets customized for local breeds.

## Materials and Methods

### Ethics Statement

Prior to their implementation, all animal procedures were reviewed and approved by the care and use committees at the United States Department of Agriculture (USDA), Agricultural Research Service (ARS) Meat Animal Research Center (USMARC) in Clay Center, Nebraska.

### Animal Samples and Genotypes

The ISGC collected and genotyped samples from 2,819 sheep from 74 breeds as part of a large study into genetic diversity and the impact of selection after domestication [15]. Samples were collected from multiple flocks to be as unrelated as possible within breed. Breeds were collected from the Americas, Africa, Asia, Europe, and the Fertile Crescent region of the Middle East where domestication of sheep is proposed to have occurred (e.g., Iran and Turkey). The geographic origin, breed identity, and number of animals per breed have been previously described [15]. DNA samples were genotyped with the Illumina (San Diego, California, USA) Ovine SNP50 Bead Array. Genotypes for SNPs were available for 2,819 sheep and extracted from this data set for analysis (Table S3).

The ISGC had selected 75 animals for WGS to extend its investigation of genetic diversity and selection in the world’s sheep breeds [10]. The majority of animals (61%) were drawn from the previous study [10] to capture the diversity present across *Ovis aries*. Additional animals were recruited that either had previously been used in the construction of genomic resources for the sheep genome [26], carried disease genes, or were wild sheep sampled from the Bighorn (*Ovis Canadensis*) and Thinhorn (*Ovis dalli*) populations of North America. Each genome was sequenced to a depth of approximately 10-fold mapped read coverage with Illumina GAII (unpublished). Prepublication access to the .bam files was provided under the Toronto guidelines for data users [27]. In total, 70 domestic sheep from 43 breed groups were used to derive genotypes for the candidate SNPs and their nearby genomic regions. These data were combined with Sanger sequence data from a U.S. panel of 96 sheep (described below) to comprise a data set from 166 sheep totaling 54 breed groups.

The USMARC Sheep Diversity Panel version 2.4 (MSDPv2.4) consists of 96 rams from Dorper, White Dorper, Dorset, Finnsheep, Katahdin, Rambouillet, Romanov, Suffolk, Texel, USMARC composite (1/2 Columbia, 1/4 Hampshire, and 1/4 Suffolk [28]), and one Navajo-Churro ram as previously described [29]. These breeds were selected to represent genetic diversity for traits such as fertility, prolificacy, maternal ability, growth rate, carcass leanness, wool quality, mature weight, and longevity. The rams sampled from each breed were chosen to minimize genetic relationships among rams within breed. These rams were also part of a set of 96 tetrad families consisting of a ram, a ewe, and twin offspring used to confirm haplotype phase of various alleles and to further evaluate the accuracy of genotype scoring USMARC Sheep Diversity Family Panel version 2.45 (MSDFPv2.45) [29]. The 96 ewes in MSDFPv2.45 consisted of 91 USMARCIII composite, two Dorset, two Suffolk, and one Romanov. DNA from these 384 reference animals were extracted by a typical phenol-chloroform-method from 3 ml of thawed whole blood previously stored at −20C [30].

Since the first report of this panel in 2010, the ram in family number 47 (USMARC Finn no. 200117718), has been reclassified as a non-family member because the genotypes from multiple disperse loci indicate it is not the sire of the twin offspring. The corresponding composite ewe (200023372) and her twin lambs (200440264 and 200440265) have also been removed. The remaining 95 families (MSDFPv2.46, Figure 4A) that continued to show proper Mendelian inheritance patterns were used for testing the accuracy, reproducibility, and segregation of MALDI-TOF MS assays for the 109 parentage SNPs.

### Four Sheep Parentage SNP Sets from other Sources

Four sheep parentage SNP sets were used in the present study (Table S4). These autosomal SNPs were derived from the Ovine SNP50k Bead Array and include: two AgResearch parentage sets (n = 84 and n = 300), the ISGC parentage SNP set (n = 88), and the CSIRO-SheepCRC parentage set (n = 382). Of the 854 members of these sets, there were 587 different SNPs.

### Criteria for Selecting SNPs Based on MAF within Breed Group

A primary consideration for selection was the SNP MAF in breeds around the world. The 47,693 autosomal SNPs from the OvineSNP50k Bead Array were screened for those that had a MAF≥0.3 in at least 36 breeds. The 0.3 threshold for MAF was chosen because it is associated with a relatively high frequency of minor homozygous genotypes (0.09 or greater if Hardy-Weinberg equilibrium is assumed). The frequency of minor homozygotes in a population is critical for parentage exclusion based on opposing homozygous genotypes. For SNPs with a MAF≥0.3, the minor allele nucleotide is often different among breeds. A simple average of the MAF among all animals leads to an inflated estimate. Thus, to correctly calculate the average MAF among breeds, the MAF must first be estimated within breed, regardless of which nucleotide is the minor allele. The minor allele for each of the 163 parentage SNPs is identified in Table S2 for each of the 74 breeds. The 36-breed threshold was used in an effort to achieve the highly informative MAF in approximately half of the 74 breeds available for study.

### Identifying nearby Polymorphisms by Analyzing WGS and Sanger Sequence

For each of the 356 candidate SNPs (Figure 1, set D), approximately 1 kb of reference sequence was extracted from the ISGC reference sheep genome assembly version 3.1. The sequences were analyzed for repetitive sequences with RepeatMasker software [31]. Nearby polymorphisms were identified in 10-fold whole genome sequence of 70 domestic sheep from 43 ISGC breed groups with software from Intrepid Bioinformatics (Louisville, Kentucky, USA) and .bam files produced by the Baylor College of Medicine (Houston, Texas, USA). Based on the relative positions of the repetitive sequences and nearby polymorphisms in these data, PCR primers were designed to amplify and sequence approximately 700 bp of genomic DNA centered on the candidate parentage SNPs that were highly informative in at least 36 ISGC breed groups and MSDPv2.4. The PCR and subsequent Sanger sequencing was carried out as previously described [32]. Candidate SNPs that could not be consistently amplified by PCR to yield a single fragment of the predicted size or give consistent clear Sanger sequencing results were dismissed from further consideration. Consensus reference genotypes for the 163 parentage SNPs for the 96 rams in MSDPv2.4 are provided in Table S5.

### MALDI-TOF MS Assay Design for a Subset of 109 Parentage SNPs

A multi-phase iterative strategy was used to validate assay development and check concordance of diplotypes derived from MALDI-TOF MS with those derived from the Ovine SNP50k Bead Array and Sanger sequencing. Prior to the development, the cutoffs for call rate and accuracy were set at 97% and 99%, respectively. Although, these cutoffs are relatively high, they are well within the capability of today’s DNA testing technology and lend substantial efficiency to testing when met. In each phase of development, the samples were blinded, scored, and decoded. Adjustments in assay conditions were made between phases of development. Genotyping was performed at GeneSeek (Lincoln, Nebraska, USA) with the Sequenom MassARRAY platform and iPLEX GOLD chemistry according to the manufacturer’s instructions (Sequenom, San Diego, California, USA). In the first phase, two multiplex assays were attempted with approximately 60 of the 163 parentage SNPs in each multiplex. The expectation was that some SNPs would not advance to subsequent rounds. Within each multiplex design, software settings were adjusted and markers grouped to maximize the number of autosomes represented, spacing between markers, and overlap with parentage SNPs from the other sources. Multiplex information and primer sequences are provided in Table S6. MALDI-TOF MS genotypes for 109 SNPs are provided for the 95 families in Table S7.

### Estimating P_I_ and P_E_ in 74 ISGC Breed Groups

The P_I_ for locus A with SNP alleles A_1_ and A_2_, was the sum of the squares of the three genotype frequencies: P_I_ =  (χ_11_)^2^+ (χ_12_)^2^+ (χ_22_)^2^, where χ_11_, χ_12_, and χ_22_ were the relative genotype frequencies of A_1_A_1_, A_1_A_2_, and A_2_A_2_, respectively [33]. The combined P_I_ for multiple SNP markers was the product of the P_I_ for each individual marker. The underlying assumption was that the marker spacing was sufficient for meiotic recombination to cause alleles to be randomly associated with one another. However, as parentage SNP density increases, the validity of this assumption is decreased. Thus, it is recognized that the combined P_I_ for 163 parentage SNPs is an underestimate of the probability of a coincidental match between random animals from the population owing to linkage disequilibrium between SNPs on the same chromosomes.

In this report, all P_E_ were estimated without the use of the other parent’s genotype information and, thus, exclusion was based only on the frequency of the opposing homozygous SNP genotypes in the offspring and the purported parent. Briefly, the probability of opposing SNP homozygotes (P_OH_) between a random offspring and a random eligible adult at SNP locus A with alleles A_1_ and A_2_, was calculated as follows: P_OH_ = (χ_11_offspring)(χ_22_adult)+(χ_22_offspring)(χ_11_adult), where χ_11_ and χ_22_ were the relative genotype frequencies of A_1_A_1_ and A_2_A_2_, respectively for the adults or offspring groups. The frequencies of homozygous SNP genotypes were assumed to be the same within a breed group regardless of age. Thus, for a single biallelic SNP, P_E_ = P_OH_ = 2(χ_11_)(χ_22_) when one of the parent’s genotypes are unavailable. This represents the fraction of eligible adults that would be excluded from parentage at one locus, averaged over all comparisons between offspring and adults. Without using the other parent’s genotype information, the combined P_E_ for multiple SNPs was as follows: P_E(SNPn)_ = P_E(SNP1)_+R_1_P_E(SNP2)_+R_2_P_E(SNP3)_ …+R_n-1_P_E(SNPn)_, where P_E(SNP1)_ represents the fraction of eligible adults excluded by the first SNP and R_1_ is the remaining fraction of unexcluded adults. R_2_ to R_n-1_ are remaining fractions of unexcluded adults after each round of subsequent testing with n parentage SNPs. Thus, for 163 parentage SNPs, the combined P_E_ for unrelated parents is given by: P_E(163)_ = P_E(1)_ + R_1_P_E(2)_ + R_2_P_E(3)_ …+ R_162_P_E(163)_. As was the case with combined P_I_, the combined P_E_ for 163 parentage SNPs is an underestimate of the probability that a random alleged parent would be excluded from parentage owing to linkage disequilibrium between SNPs on the same chromosomes. For related parents, the P_E_ for each SNP was multiplied by a coefficient of relatedness (*r*), where *r* = 0.125, 0.250, or 0.500 [34]. Thus, P_E(163)_ for related parents = (*r*P_E(1)_+*r*R_1_P_E(2)_+*r*R_2_P_E(3)_ …+*r*R_162_P_E(163)_).

## Supporting Information

Figure S1 **Distribution of SNP informativity in ISGC breed groups.** The MAF was calculated for 47,963 autosomal SNPs in the Ovine SNP50k Bead Array for each of the 74 ISGC breed groups. SNPs with a MAF greater than or equal to 0.3 in an ISGC breed group were defined as highly informative in that group.
(TIF)

Table S1 **Features of 163 sheep parentage SNPs.**
(XLSX)

Table S2 **Statistics for allele frequency, P_I_, and P_E_ for each 163 sheep parentage SNPs in breeds from around the world.**
(XLSX)

Table S3 **OvineSNP50k Bead Array genotypes for 2,819 ISGC sheep and 163 parentage SNPs.**
(XLSX)

Table S4 **List of 587 SNPs from four parentage sets.**
(XLSX)

Table S5 **Consensus reference genotypes for 96 U.S. sheep (MSDPv2.4) and 163 parentage SNPs.**
(XLSX)

Table S6 **Oligonucleotide sequences for multiplex MALDI-TOF MS assays of 109 parentage SNPs.**
(XLSX)

Table S7 **MALDI-TOF MS genotypes for 380 U.S. sheep (95 families, MSDFPv2.46) and 109 parentage SNPs.**
(XLSX)
