# Supplementary figures and images for: SNPs for Parentage Testing and Traceability in Globally Diverse Breeds of Sheep
Source: PLoS One. 2014 Apr 16;9(4):e94851. doi: 10.1371/journal.pone.0094851 (PMC3989260; doi:10.1371/journal.pone.0094851)

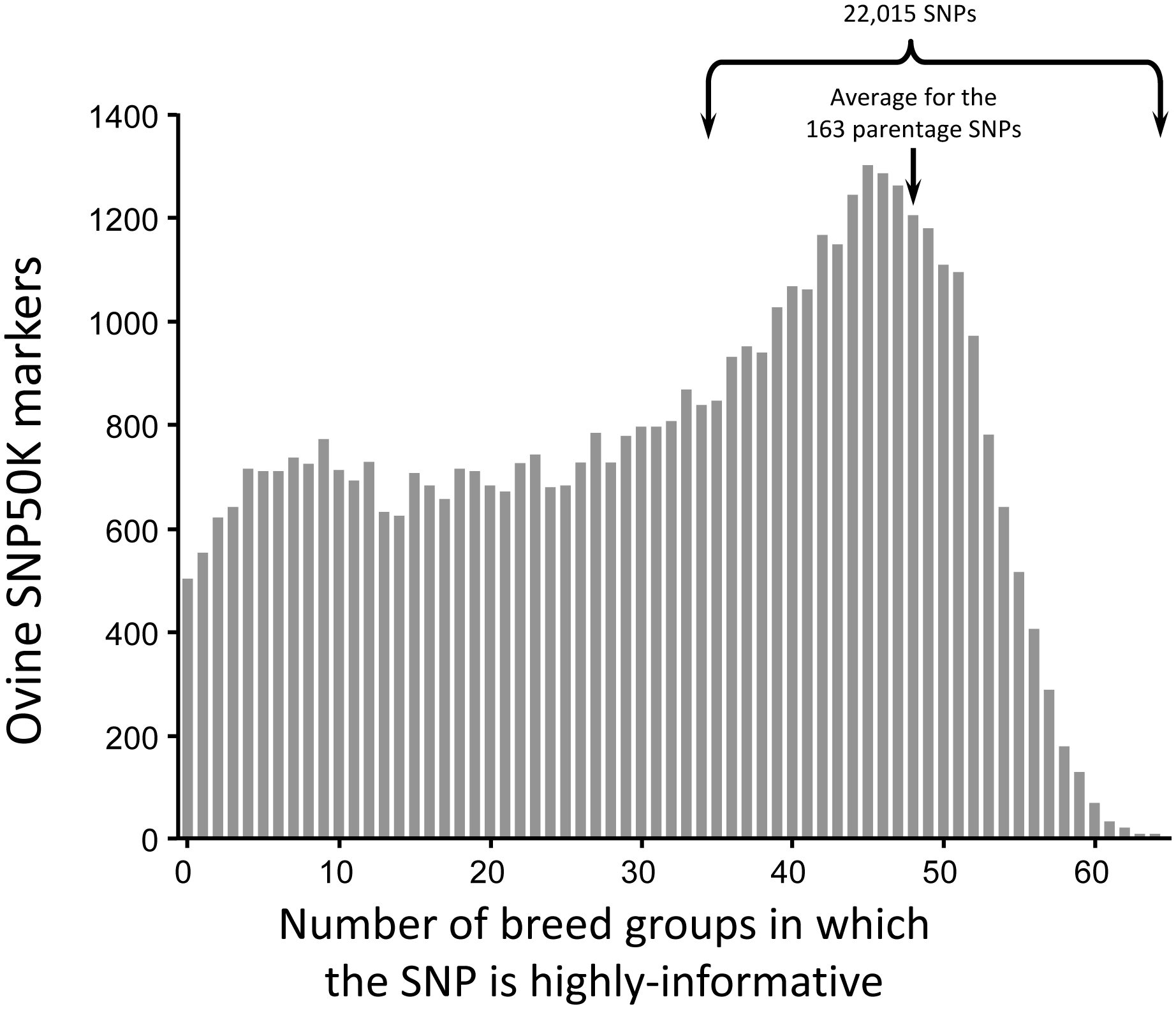

Supplement: Figure S1 — Distribution of SNP informativity in ISGC breed groups. The MAF was calculated for 47,963 autosomal SNPs in the Ovine SNP50k Bead Array for each of the 74 ISGC breed groups. SNPs with a MAF greater than or equal to 0.3 in an ISGC breed group were defined as highly informative in that group. (TIF) [file pone.0094851.s001.tif]
